# A qualitative exploration of counterfeit, substandard, spurious, and adulterated drugs in Pakistan: A perspective of drug law experts

**DOI:** 10.1371/journal.pone.0322188

**Published:** 2025-04-24

**Authors:** Farooq Bashir Butt, Hamid Saeed, Zobia Mubarak, Syed Atif Raza, Usman Rashid Malik, Furqan Khurshid Hashmi

**Affiliations:** 1 Punjab University College of Pharmacy, University of the Punjab, Allama Iqbal Campus, Lahore, Pakistan; 2 Primary and Secondary Healthcare Department, GOVT of the Punjab, Lahore, Pakistan; 3 School of Pharmacy, Xi’an Jiaotong University, Xi’an, China; 4 Faculty of Pharmacy, University of Lahore, Lahore, Pakistan; University of Georgia, UNITED STATES OF AMERICA

## Abstract

**Objectives:**

The issue of quality of medicine is a worldwide phenomenon and counterfeit, substandard, spurious, and adulterated (CSSA) drugs are a substantial threat to public health. This issue is rampant in the context of low-middle-income countries such as Pakistan. The current study involved a phenomenology-based qualitative approach to explore these drugs’ perception, knowledge, practice, and issues in combating this menace.

**Methods:**

A semi-structured interview guide was developed. Eleven drug law experts were interviewed through a purposive sampling technique. All interviews were audio recorded, transcribed verbatim, and analysed using a framework analysis approach, yielding seven distinct themes.

**Results:**

The results showed that CSSA drugs are a serious public health threat and drug law experts confirmed its prevalence in the market. They indicated shortcomings in legislation up to the extent of undue amendments, failure to interpret and implement the law by regulators, ineffective law enforcement machinery, the sub-optimum performance of quality control boards, drug testing laboratories, and courts, and the dubious role of rogue middlemen and wholesalers in drug supply chain and corruption were salient issues highlighted.

**Conclusion:**

The study revealed that proper drug surveillance, ensuring the presence of a pharmacist, enforcing the law, securing the supply chain, infrastructure for a drug control regime, and training regulators can help tackle this problem.

## Introduction

Counterfeit/spurious, medicines are on the rise all around the world however, this issue is more serious in lower and middle-income countries (LMICs) [1,2]. Pakistan is a developing country where this public health problem is more intricate, as a majority of the drug stores have staff with little or no professional training and most of the drug sellers have fragmentary knowledge regarding dispensing and storage [3]. Coupled with an unregulated segment of unauthorized wholesale dealers/middlemen exploiting loopholes in the system.

In 2016, according to data from one of the drug testing laboratories in Pakistan, 43,705 samples were sent to drug testing laboratories and 96 (0.22%) of them were found spurious. In the year 2017, as many as 59,611 samples were collected and the laboratory declared 83 (0.14%) of the samples as spurious. In 2018, 44,146 samples were collected and 41 (0.09%) were found spurious. However, in 2019, only 24 (0.06%) samples out of 41,700 were found spurious [4]. The data mentioned the results of one of the public sector Drug Testing Laboratory analysis which tested drug samples sent by provincial inspectors of drugs in the fields and from the public sector hospitals where all the tender business medicines are mandatorily tested before issuance to the public. Moreover, the sampling method is not based on a risk-based random sampling technique. So, this data is not a true representative of the prevalence of CSSA drugs. A drug misadventure in 2011–2012 at the Punjab Institute of Cardiology (PIC) Lahore, claimed more than 200 lives wherein cardiovascular drug (Isosorbide mono-nitrate) contaminated with anti-parasitic drug pyrimethamine was consumed by patients, which underpinned the need for in-depth research [5,6].

In 2004, the World Health Organization (WHO) alleged that 40–50% of drugs consumed in Pakistan were counterfeit or substandard. Similar figures were given for the prevalence of substandard and spurious drugs [7–9]. The general public including physicians and journalists enquired, creating doubts about the quality and efficacy of pharmaceuticals which might be due to disparity of price among the same generics, lack of knowledge, and unknown reasons [10].

There is no universal definition of counterfeit or spurious drugs and legal definitions vary from country to country. The lack of a uniform and standardized definition has become a hurdle in fighting counterfeit drugs [11,12]. Therefore, it is challenging for all countries to have adequate legislation for dealing with counterfeit drugs [13]. Counterfeit medicine was defined by WHO as, one which is deliberately and fraudulently mislabelled concerning identity and/or source. Counterfeit can be both branded and generic products, which may be a product with the correct ingredients, wrong ingredients, without active ingredients, with insufficient quantity of active ingredients, or with fake packaging [14].

Pakistan’s Drugs Act 1976, defines ‘counterfeit’ as a drug or any other substance or preparation or any homeopathic, Unani (Greek), ayurvedic or biochemical medicine or any other preparation offered for treatment or prevention of any disease, the label or outer packing is a copy of or looks like as to be reckoned to cheat the label or outer packing of a drug of another pharmaceutical producer. Adulterated means a drug which consists in whole or part of any filthy, putrid, or decomposed substance or which contains any foreign matter, vermin, worm, rodent, or insect or which has been manufactured under unsanitary conditions or the container releases any poisonous or deleterious substance which may render the contents injurious to health. In contrast, substandard means a drug that is not of specifications. A spurious drug has been defined as a drug that does not contain the active ingredient, and the manufacturer is either fictitious or does not exist at all [15]. However, in May 2017, the WHO, introduced the term substandard and falsified (SF) medical products replacing the “counterfeit” term to bring more clarity and uniformity/harmony and to coin a broad-based definition for drugs of dubious quality [16].

The WHO estimates that counterfeit drugs may comprise approximately 10% of all drugs manufactured globally while less than 1% of drugs in industrialized countries are spurious, in certain third-world countries, it may be up to 60% [12,17]. Spurious drugs are regarded as 5–7% of the drugs spread in the EU and may constitute up to 15% [18]. Moreover, the Centre for Disease Control and Prevention (USA) has estimated that the percentage of spurious drugs in industrialized countries is between 1% and 10%, and maybe 30% in countries in Africa, Asia, and Latin America [19,20]. Similarly, the United States Food and Drug Administration(US FDA), consistent with the WHO has estimated that spurious drugs constitute 10% of the global drugs market; however, of that, only 1% or less are sold in the US market [21]. Theoretically and ideally drug supply chains represent that the pharmaceutical firms distribute their medicines straight to leading distributors/wholesalers. These Distributors/wholesalers are authorized agents of the pharmaceutical manufacturers, ship the medicines right to retail community pharmacies or healthcare settings, which then pass out these drugs to the ultimate consumers**/**patients. However, in the real world, the pharmaceutical supply chain is both complex and long where drugs pass through multiple transactions, going back and forth, before reaching the supply point. The risk of counterfeit/spurious medicines reaching patients and consumers increases with the increasing complexity of the supply chain [22,23].

Pakistan is beset with similar issues, intrigued with multiple levels of wholesalers. SF drugs reach the consumer, ultimately through this long chain of unauthorized wholesalers coupled with weak regulatory oversight and infrastructure are the salient issues. Previous studies about this topic in the context of the Pakistani system are scanty and did not address the issues related to combating CSSA drugs. The present study aims to explore and highlight the issues in combating CSSA drugs in Pakistan so that effective policy may be developed to overcome the increasing menace of such drugs in Punjab as well as in the whole of Pakistan.

## Methods

A phenomenology-based qualitative methodology was used as the study design as it offers an in-depth exploration of respondents’ knowledge, experiences, and perceptions. A semi-structured interview guide was designed to explore the perspectives of the interviewees in connection with their perception of counterfeit, substandard, spurious, and adulterated (CSSA) drugs and issues encountered in combating this menace. The interview guide was prepared based on the literature review and current prevailing practices in Pakistan. It was reviewed using argumentative and cumulative techniques and subjected to validation and reliability assessment before data collection. The guide was pilot-tested on two participants (Drug Law experts/Drug Lawyers) and changes were made accordingly [24]. There was no major change suggested by the interviewers it was only related to the structure of the guide and length of questions so they were rectified before making the guide available for the final interview. Interviews were conducted in a separate area where the chances of interruptions were minimal, and where in-depth, freely expressed discourse was possible.

### Participant and inclusion criteria

A purposive sampling method was adopted to select senior and experienced Drug Law experts (lawyers/advocates) with vast drug case experience. Some of them were both pharmacy and law graduates. The participants consented to participate in the study and were also given explanatory notes to describe the aims and objectives of the research.

### Ethics approval

The study was approved by the Institutional Review Board (IRB), University of the Punjab, Lahore, Pakistan (Letter No: D/191/FIMS).

### Study setting

The study was mainly conducted in Punjab, Pakistan which is the largest province of the country comprising 56% of the total population of Pakistan. The focus of the study was drug regulation prevailing in Punjab, therefore, the drug law experts mostly belonged to metropolitan cities of the Punjab. Moreover, Punjab is the hub of pharmaceutical manufacturing, having a wider infrastructure of drug supply chain, connecting community pharmacies and health care establishments.

### Exclusion criteria

General Advocates without any relevant experience in drug cases were excluded from the study.

### Data collection and processing

Participants were recruited in the third week of April (from 16^th^ of April – 13^th^ May),2022 using a purposive sampling technique and interviews were started in the first week of Jun 2022. After getting the written consent to participate, the researcher (FB) arranged a meeting with the participants at their convenience at the place of their choice. Saturation was reached at the 9^th^ interview; however, two additional interviews were conducted to confirm the saturation. Saturation was determined by applying the saturation determination method for qualitative study as reported [25,26]. Interviews were conducted in English as all respondents could converse in English. Each interview lasted for approximately 45 minutes. All audio-recorded interviews were transcribed verbatim. The interviews were attested for accuracy and consistency by listening to the recordings time and again. The authors analysed the written text and transcript word by word, which were read repeatedly to confirm accuracy and robustness. The whole transcript was thematically analysed for content and emerging themes.

### Data analysis

The framework analysis approach was selected as the most appropriate. This technique is used for data analysis in primary qualitative research.; framework analysis is a matrix-based method involving the construction of thematic categories into which data can be coded [27]. This approach was found suitable for present research as it contained specific questions, a defined and limited timeframe, a sample that is predesigned, and *a priori* issues identified from the outset as requiring addressed [28]. Nevertheless, researchers (FB, FH, and HS) scrutinized the data from the transcripts of the interviews by isolating and identifying the emerging themes from the interviews. These themes were useful in forming a theory relating to issues involved in combating CSSA drugs from the perspective of drug law experts. The themes were identified manually from the data by thoroughly reviewing the transcripts of the interviews, and sub-themes were also generated, which were checked by the team of the research group.

## Results

The present research included eleven senior drug law experts who had different levels of experience. There was only one female (n=1) among the respondents. The majority (n=7) of respondents were from the age group of 50–60 years or above, and three were in the age group of 41–50 years. Eight Drug Law experts had working experience of 20–40 years and were engaged in legal representation of the firms in different regulatory bodies and Courts as shown in Table 1.

**Table 1:** Demographic characteristics of drug law experts (N=11).

Characteristics	n (%)
**Gender**	
Males	10 (90.9)
Female	1(9.1)
**Age (years)**	
30–40	1(9.1)
41–50	3(27.2)
51–60	4(36.3)
61–70	1(9.1)
71 or above	2(18.2)
**Educational status**	
Bachelor of Laws (LLB)	11(100)
**In addition to a law degree**	
Master of Laws (LLM)	2(18.2)
B. Pharm/PharmD	3(27.2)
Master (Pharmacy)	1(9.1)
Master (others)	3(27.2)
**Experience (years)**	
6–10	2(18.18)
11–20	1(9.09)
21–30 or above	8(72.72)
**Number of cases in a month**	
11–20	3(27.27)
40 or above	8(72.72)
**Status of legal experts**	
Advocates	7(63.63)
Advocates/ Drug laws consultant/ Regulatory Manager with Pharmacy degree	4(36.36)

The study results started by exploring the drug law experts’ views about counterfeit, substandard, spurious, and adulterated drugs and further asking which one of them was most dangerous and harmful. Participants also furnished views on the sufficiency of the existing laws and the role of regulatory authorities in this context. The role of media and the perception of the common man on the issue were also discussed at length. In the end, the views of participants about drug testing laboratories, regulating bodies like quality control boards and courts, the way forward, and expected outcomes from the policy change were discussed. During the analysis, seven major themes emerged as shown in Table 2.

**Table 2:** Responses of drug law experts (DLE).

No	Themes	Responses
1.	**Awareness of the terms CSSA**	*Counterfeit is “copy” and it is not a drug…. counterfeit is packing to deceive and there is no difference between original and copy…but outwardly it looks to be the same LE1* *The counterfeit drug in common language is a drug that gives a cheating effect from the outer label or labelling or outer packing in such a way that it resembles very closely …packing of a drug of another manufacturer LE 4* *Mostly in cases of non-drugs or Unani, etc., allegations of counterfeiting of popular allopathic drugs have been made as a Unani or homeopathic or non-drug could not be deemed to be a drug or spurious, but it copies LE 10*
2.	**Quality of medicines and the current system of regulation**	*A licensed manufacturer never manufactures spurious drugs because he has made investments. His whole reputation would go into “minus” by one spurious drug…who manufactures spurious drugs? Middleman…there are examples…. However, sometimes manufacturers of spurious drugs put 10% active ingredient so it would be substandard…” LE 1* *“I believe that any manufacturer will not deliberately manufacture or sell any suspected batch of a drug as it will not only harm his reputation, prestige, and standing in the market as well as comity of manufacturers but will also be counterproductive as in the event of any negative test reports or prosecution, etc. he could suffer beyond his imaginations including fines, imprisonment, deregistration of drugs and suspension or cancellation of the drug manufacturing license which is manufacturer’s basic source of income.” LE 10* *“According to WHO reports…spurious medicine. In China more than 3% and in India more than approximately 2% and in Pakistan, not more than 1% …. Less than 1%...” LE 3* *There is a major issue of substandard drugs ……Quality of drug may suffer due to some mechanical or human error during manufacturing or due to poor storage conditions at the distributor or retail pharmacy level” LE 11* *“…So far the regulatory authorities of the healthcare system have failed to play their effective role to eradicate the menace in this field” LE 4*
3.	**Role of media to combat counterfeit, substandard, spurious, and adulterated drugs**	*The role of media is positive in the PIC (Punjab Institute of Cardiology) case. If the media did not raise hue and cry, chances of more deaths were there” LE 1* *“It is a double edge weapon…it may be used in both ways” LE2* *I do not feel any role is to be played by the media for positive or negative…improvement in the field of drugs generally as it should be done and is the prerogative of the stakeholders alone. Only the publication and availability of the drugs with the permission and approval of the authorities should be permitted through the media and nothing more.” LE 10* *“It is the work of media to collect information and then publish it accordingly. The role of the investigator, prosecution, and the court is to thrash out the facts from fiction without considering the media reports” LE 5*
4.	**Shortcomings in laws, rules, and regulations**	*“I believe there is more emphasis on punishments rather than on trying to achieve the target of having the best quality products…. based on the threats and fears of the manufacturers and dealers of having to undergo the trials and tribulations of cases, fines, penalties, etc. which fear are unfortunately cashed and abused by the unscrupulous elements of society” LE 10.* *“If one tablet of the unregistered drug is recovered from a peddler is equivalent to 100000 tablets recovered from the manufacturing, the penalty is same for both, which is against the laws of natural justice….in my view, section 27 should have a quantum, scale, and sentencing regarding the quantity, nature, heinousness and other circumstances of the drugs.” LE 5* *“There is no specific time limit …mentioned in the Drugs Act 1976 to prosecute/ take action against the culprit. Even in cognizable cases, there is a very complicated procedure for registration of case as the show cause/personal hearing is mandatory; therefore, it creates a delay in launching of First information report (FIR)/prosecution against the culprit…” LE 6* *“PQCB must be independent comprising of maximum five members…. no employee of health department should be its member and chairman must be retired judge of High Court” LE 1*
5.	**Quality of analysis from government laboratories**	*Provincial labs must make sure that standard quality medicines are available in the market and then in case of any difference of opinion or case of retest, the appellate lab has the same function to ensure that whether the sample drug is of standard quality or not. ……it is to be ensured whether the sample drugs were stored properly or not. If it is not stored properly, then there may be variations of results of test reports that are only due to storage conditions LE 2* *“During my practice, I have faced many problems regarding DTL reports, mostly reports are incomplete and do not provide any admissible evidence during the trial.” LE 8* *“Many times, reports of laboratories are time-barred and there are no extensions obtained from quality control Board” LE 11* *The actual problem is that they don’t have medical devices, biological testing, or herbal nutraceutical testing labs…they are very poor in testing…I am not satisfied…. Fully skilled and well-trained certified staff must be appointed… they are experimenting, even Drug inspectors also doing experiments, so it is a criminal negligence… army without training” LE 9* *“. Due to extreme corruption, drugs are declared as substandard ……drug inspector is appointed as analyst and that hapless does not know how to conduct analysis…” LE 1*
6.	**Role of Quality Control Boards and Drug Courts**	*It is the legal requirement that before trial the matter should be referred to the provincial quality control Board or DQCB for scrutiny and the duty of the concerned Board is that he must check whether the proceedings of Drug Inspectors are according to Law and procedure or not but unfortunately, they played the role of the post office and there is no such scrutiny which is provided by law and that is why there is the little ratio of conviction in drug cases during trial.…” LE 2* “*Standard of scrutiny…not satisfactory …. Sometimes we are feeling just friendship club of PQCB members, obliging the people” LE 9* *Only a few cases were decided against the accused and the court convicted the accused in rare cases, the most accused were acquitted in these cases because of defective investigation, insufficient evidence, and delay in legal formalities done by the drug authorities.” LE 8* *“….in some cases, due to lack of evidence or poor evidence, as the Drug Inspectors while under examination tends to answer which ultimately give benefit to the accused. The investigator must collect evidence beyond the shadow of reasonable doubt. So, courts are rightly deciding the cases on their merits.” LE 5* *“The accused acquitted from the Drug Courts based on some serious lacunas left by the Drug Inspector or relevant Board in the case, i.e., violation of mandatory provisions of Drug Act, (i) Gazette Notification (ii) Time barred DTL report (iii) not making the responsible person as an accused person (iv) violating the principle of safe custody, i.e., not producing the case property in the court (v) defective investigation (vi) Not mentioning time, date and storage condition in seizure form. LE 6* *“Chairman of Drug Court is required to be a judge of the high court, preferably retired, in consultation with the Chief Justice of the concerned high court and members must be independent… not part of the executive” LE 1*
7.	**Provision of training and supply chain issues**	“...*training is sin-qua-non…” LE 5* *“…training is essential…. more than you think. regulators should know this book (Drug Laws) which is not there….” LE 1* *“…training is needed regularly…as pharmaceutical legislation worldwide is rapidly changing both on the technical and legal side...LE 7* *“…As per section 32 (Drug Act 1976) the manufacturer will give the drug to his agent who is a middleman and the agent will supply for sale and not for resale…. now the question is who manufactures spurious drug? (pause)...middleman…there are examples…LE 1* *“…. whenever substandard and spurious drug is intercepted the wholesale dealers in most of the cases are involved …. but in some cases, they join hands with drug inspector” LE 2* *“The Drug Act 1976 provides clear provision under section 32(3) that a manufacturer of the drug and his agent thereof for distribution has no defense in any contravention under the Drug Laws so both are same and liable for each other under the rule of strict liability…” LE 5* *“Mostly wholesalers and middleman are responsible for substandard and spurious drugs…the chemists are duty bound to buy medicines from the manufacturer or its authorized distributors …. but regulators do not know…” LE 6* *“Medicines are substandard due to supply chain issues, including distributor and sub-distributor wholesaler. In Pakistan, provincial governments are not fully equipped to regulate sub-distributors or the wholesaler……inefficiency of regulators has contributed much to the spread of this menace…. LE 7*

### Theme 1: Awareness of the term CSSA

Most of the participants explained their understanding of these terms, which are frequently used in our context. All drug law experts shared their understanding of these terms and their injurious effects on society. They mainly emphasized that counterfeit is not related to the content of the product rather it relates to outer packing. Without any exception, all the participants considered manufacturing and selling counterfeit/spurious drugs as the most heinous crime against society and a threat to public health. The majority of the key informants believed that substandard drug was the major issue prevailing in society.

### Theme 2: Quality of medicines and the current system of regulation

All the participants confirmed the availability of these drugs in the market, but their perceptions were a bit different from each other based on their practical experience. Participants perceived that spurious drugs were not manufactured by licensed manufacturers as the stakes were very high.

Similarly, participants pointed out that substandard drugs are highest in numbers among all the categories as compared to adulterated and spurious drugs which could be due to inadvertent mistakes in Good Manufacturing Practice (GMP) mechanical fault or due to poor storage conditions or due to faulty report of analyst because no licensed manufacturer deliberately manufactures the substandard drug.

### Theme 3: Role of media to combat counterfeit, substandard, spurious, and adulterated drugs

According to some participants, the media had been blamed for spreading sensational news to increase its circulation. The majority of the participants gave mixed views about the role of media and blamed it for spreading rumors and faux news. Some considered it to be a prerogative of stakeholders and the job of media was just to collect information and then publish it as such, accordingly. However, in the Punjab Institute of Cardiology, Lahore drug fiasco in 2012, where more than 200 people lost their lives for ingesting tainted/contaminated cardiac drug Iso tab (Isosorbide), the role of media was appreciated because it created hue and cry and government functionaries became active to identify the root cause of this debacle which resulted in minimizing any further human loss.

### Theme 4: Shortcomings in laws, rules and regulations

Most of the participants drew attention to the various shortcomings in the law, rules, and regulations. Some of the participants believed that harsh punishments under the Punjab Drugs (Amendment) Act 2017&2018, were not practicable and merely a source of corruption for regulatory officers who misused their authority in the guise of such punishments. One participant observed that there was more emphasis on punishments and fines instead of trying to bring any substantial improvement to the system.

They highlighted various blatant flaws in the legislation. It was also mentioned by one of the participants that penalties provided in law were not logical as neither was the quantum of punishment given in section 27 of the Drugs Act 1976 nor was there a proper sentencing for the same. The punishment for recovery of one unregistered tablet or a full batch of the same tablet is the same which is against the spirit of the law Table 2.

### Theme 5: Quality of analysis from government laboratories

Some of the participants highlighted glaring mistakes in the reports of Government Analysts, especially the drug law experts having a background in Science/Pharmacy, etc. They pointed out that newly appointed pharmacists (staff) were novices and directly embarked on testing drug samples without any regular training in analysis. They further elaborated that the reports of analysts were incomplete and failed to provide admissible evidence about the quality of the drug sample tested. The participants also explained their perception of variation and anomalies in reports of different laboratories of the same batch of drugs. The benefit of the doubt due to the discrepancy in reports of different laboratories is always extended to the accused party.

### Theme 6: Role of Quality Control Boards and Drug Courts

One participant stressed the need for establishing an independent Provincial Quality Control Board (PQCB) under the chairmanship of a retired Judge of the High Court. It was a legal requirement that all the cases must be scrutinized under section 11(5) (b) (e) of the Drugs Act 1976.

It was the unanimous opinion of participants that Boards played the role of just a post office and forwarded the case to the Court without proper scrutiny and application of an independent judicious mind which many times resulted in the acquittal of the real culprit. They showed their dissatisfaction with their work and declared them a friendship club, obliging the criminals by involving innocent people.

They further elaborated that Mala-fide in investigation cannot be ruled out as everyone needs money. The participants affirmed that the drug court could not sentence an accused on the weak case of prosecution. If there are inherent defects/lacunas in the case, the benefit goes to the accused as the prosecution has to stand on its legs. One of the participants however confirmed that drug court never spared a case of spurious drug even if it had some procedural deficiencies against an innocent person.

Participants highlighted the need to present strong cases before the Court for trial so that criminals were awarded exemplary punishments. This is not possible if the prosecution case is frail and there are incurable irregularities and infirmities in these cases, both on the part of the drug inspector and regulatory bodies like Quality Control Boards.

Most of the participants were of the view that drug inspectors failed to investigate drug cases properly. Investigation conducted by law enforcement agencies like police/ federal investigating agencies (FIA) is flawed as they don’t have people with the required technical knowledge. They lamented the attitude of the drug inspectors being casual and irresponsible.

All the drug law experts were of the view that the collection of evidence is the primary task of a drug inspector for which they were not given any proper training by the authorities. The defects in investigations ultimately give benefit of doubt to the accused.

### Theme 7: Provision of training and Supply chain issues

The majority of the participants emphasized the need for training of stakeholders especially regulators and Government analysts for effective prosecution of drug cases of substandard and falsified (SF) drugs. They elaborated that the world was changing as well as pharmaceutical laws. To overcome regulatory challenges, training is mandatory to optimize their performance and combat the menace of substandard and spurious drugs.

Similarly, participants of the study vehemently criticized the role of the middleman in the drug supply chain. They agreed that spurious drugs were not manufactured by licensed manufacturers rather it was the middleman who was the real culprit. The middleman mixes spurious drugs of the same batch number with standard quality drugs of licensed manufacturers, hence, shifting the liability of SF drugs on that manufacturer.

Some of the participants maintained that regulators had either meager knowledge of drug laws or due to the wrong interpretation/ignorance of the law, the main person involved in the spurious drug business was not implicated as accused in the majority of cases. In this way, the main culprits escape the clutches of law. One participant opined that the provincial government was not fully equipped to regulate a sub-distributor or a wholesaler. They further explained that a warranty was the mechanism of track and trace but would only be valid if issued by the manufacturer, importer/indenter, or their authorized agent.

## Discussion

The main objective of the research was to explore the knowledge, experience, and opinions of drug law experts concerning issues encountered in combating CSSA drugs in society. There was a paucity of information from Pakistan, and it was a new experience. The framework approach of data analysis was employed in identifying the salient themes emerging from the data. The study generated various findings which were grouped into seven main themes which would lay down the foundation for a comprehensive strategy and campaign to address the main causes of substandard and falsified drugs for policymakers.

The findings of the study showed that all the participants confirmed the presence of CSSA drugs in the market, but it was not believed to be manufactured by a licensed manufacturer which seemed to be a reasonable stance. The participant considered spurious drugs as the most dangerous and one of the most heinous offenses in the world. The risk involved to the common man from spurious drugs was highlighted by the respondents because such medicines were manufactured and distributed in the absence of any regulatory oversight of any authority. There are several studies regarding injuries and deaths to patients that are linked to the consumption of counterfeit medicines. In 2005, more than 1000 were hospitalized in Russia, and 50000 people were inoculated with a fake meningitis vaccine in Nigeria, resulting in the deaths of 2500 children in 1995. The prevalence of CSSA drugs is a serious public health hazard resulting in increasing morbidity and mortality, adverse drug reactions, therapeutic failure, and a rise in drug-resistant pathogens. Similar views were expressed in a report by WHO published in 2006, which stated that counterfeit drugs were becoming a public health crisis worldwide [29].

Some of the participants considered substandard and adulterated drugs as a result of accidental or industrial failure rather than a deliberate offense committed by the pharmaceutical manufacturer. This view is quite plausible and demands that the intent of the manufacturer must be considered before proceeding against him. According to some participants, spurious drugs are intentionally manufactured and are heinous crimes against society. However, counterfeit drugs are lawfully manufactured and mostly of standard quality, but their packaging resembles some other famous drug. In this connection, continuous surveillance and repeated inspections conducted by trained inspectors, who have experience in GMP audits of pharmaceutical firms, are the solution. Participants highlighted the limited resources and infrastructure, lack of facilities and logistic support extended to drug inspectors in the province, the dubious role of media, missing community pharmacists link, unregulated middlemen/wholesalers in the drug supply chain, defective reports of drug testing laboratories, lack of proper scrutiny of drug cases at Quality Control Boards level, decisions of drug courts based on lacunae and material loopholes, shortcomings in legislation, poor interpretation/implementation of the law, misuse of authority on the part of public functionaries in the garb of harsh punishments and corruption at various tiers in the system without any exception were major issues involved in combating CSSA drugs.

The participants were found to identify a very crucial role, that pharmacists could play in combating CSSA drugs by purchasing medicines from manufacturers or other reliable sources like authorized agents, which were also found in some of the literature [10,20,30–34]. Similar views were expressed, highlighting the role of Pharmacists not only to provide quality extended pharmacy services but also quality medicines [35,36]. In another study, the proportion of licensing requirements was 19.3%, with a few qualified persons (22%) [37], which showed the dismal state of affairs as sales of drugs were in the hands of uneducated people. It was a threat to public health and unheard of in the developed world where the pharmacy would close in the absence of pharmacists.

The absence of pharmacists is the fault of drug inspectors for not ensuring the availability of pharmacists at the pharmacy, which is a statutory requirement and is also one of the major impediments in combating CSSA/SF drugs from society. It also results in depriving the community of not only quality medicines but also extended pharmacy services as health is recognized as a fundamental right leading to quality of life. The findings also showed that regulatory authorities lack professional competence, legal acumen, training, political will, and even integrity to combat the menace of substandard and falsified drugs in low- and middle-income countries like Pakistan.

The study found that unnecessary amendments in law were not warranted instead of focusing on the implementation of drug law which was a real challenge, as merely enhancing the punishments was not the solution to the problem. Harsh punishments and exorbitant fines are a source of corruption, as regulators are often blamed for misusing their authority. Similar results were mentioned in a study where corruption in the procurement of pharmaceuticals and weak legislation was considered the root cause of the menace [38]. Another study, conducted in Pakistan, identified factors that caused the spread of counterfeit drugs as a lack of law enforcement, low consumer awareness, and weak legislation [39].

Defining counterfeit drugs has been a challenging job and it has been thought that lack of a uniform and standardized definition for such drugs has become a hurdle in fighting counterfeit drugs” [11,12]. In Pakistan, the term spurious drugs is more common than counterfeit drugs and is considered a public health threat. Therefore, it is challenging for all countries to have adequate legislation for dealing with counterfeit/spurious drugs [13]. The layperson in the country is not aware of the difference between counterfeit/spurious or substandard drugs due to a low literacy rate and common public perception. Now the term substandard and falsified (SF) is an internationally accepted term, used for all drugs with questionable quality [40].

Findings also showed that our government was not serious about controlling substandard and falsified drugs but more interested in point scoring through cosmetic actions and lip service. The policies made were based on assumptions and surmises rather than ground realities and evidence. It was also highlighted in research that the role of media was mostly unscrupulous which portrayed generic, herbal medicines as counterfeit/spurious drugs without any evidence. It maligned the local pharmaceutical industry and hampered the export potential of pharmaceuticals to global markets which was a disservice to the country. However, in one study in Nigeria, media use proved to be positive and resulted in a substantial decline in counterfeit drugs [41]. Physician in Pakistan were also found declaring a drug counterfeit/spurious to their patients on their whims if the brand prescribed by them wasn’t available and substituted, merely for vested commercial interest. Patients have blind trust in physicians and they get their medicines checked by them before usage on the asking of physicians under the influence of unethical marketing practices. Such practices resulted in tarnishing the image of pharmaceuticals in the eyes of the common man. However, a study in Pakistan highlighted various steps taken by the Punjab Government, inter alia; IT-based interventions in Punjab to bring paradigm change in the drug control regime including the revamping of drug testing laboratories, provincial quality control boards, medicine surveillance system-centralized dashboard, drug courts, centralized drug sale licensing (CDSL), and 2D barcoding serialization system for public procurement [42,43].

The research emphasized the need for training of not only drug inspectors but also drug analysts and other stakeholders to optimize their performance to enable them to properly interpret and implement law for effective prosecution of cases and robust testing in laboratories which was the requirement of law. In this connection, the establishment of a Centre for Professional Development (CPD) of pharmacists for capacity building is the need of the hour.

The study found that outcomes of the study were likely to change people’s and policymakers’ behaviour to identify real issues involved in the war against substandard and spurious drugs. One of the studies that was repeatedly quoted in the literature in our context was about the testing of 96 samples of ceftriaxone injections from 33 pharmaceutical manufacturers in Pakistan. It showed that since the nature of drugs cannot be ascertained by the naked eye or by everyone, the study randomly selected 96 samples of different strengths of Injection Ceftriaxone Sodium and its generic (a widely used third-generation cephalosporin) in Pakistan were tested. Out of these, 15.62% of Ceftriaxone Injections were found to be “out of Specification”; however, no sample was found fake (spurious) out of all the 96 samples tested [10]. This study corroborates that spurious drugs are not more than 1% in the market, and the major issue lies in substandard medicines. These results are contradictory to the WHO report, which seems to be based on media reports and is factually incorrect.

The excessive punishments introduced through amendments in drug laws were a source of corruption rather than to curb the menace of CSSA drugs. This was also corroborated by Harris et al who maintained that harsh punishments may be associated with an increased risk of drug counterfeiting where organized criminals develop corrupt relationships with law enforcement agents [44]. Moreover, there was no mention of the quantum of punishment for recovery of one tablet or large quantity of unregistered drugs. The sale of public sector hospital medicines/government property medicines “not for sale” was not an offense of drug law, which were declared as without warranty by inspectors, warranting the attention of legislators. Moreover, the timeframe for prosecuting/launching of case was also a missing entry which required the attention of legislators and policymakers. Similarly, the establishment of a special unit of police or separate police stations for drug cases under pharmacists for effective prosecution and convictions seems to be a plausible suggestion in the circumstances.

The most important finding identified in this research was the “porous and vulnerable drug supply chain” having a long chain which also included unauthorized sub-distributors infiltrating the legitimate drug supply chain at various points. Poor regulatory oversight and non-implementation of drug laws were the salient factors mainly responsible for the spread of this menace. The traceability of pharmaceuticals is obfuscated and difficult to identify in our context, as criminal counterfeiters are exploiting the loopholes in the system, enhancing their false profits due to regulatory gaps, at the expense of patient health [45]. In this regard, the Drug Court Lahore handed down a detailed landmark judgment which was upheld by the Lahore High Court, about “warranty”/ pedigree which was a track and trace system [46]. It was held by the court that the drug manufacturer and his/her agent for distribution had no defense in any contravention, as mentioned in section 32(3) of the Drugs Act 1976, and under the rule of strict liability as a wholesale distributor was held as part of the manufacturer. It was found that most of the regulator’s concept about warranty in the drug supply chain was not clear due to poor interpretation and understanding of the law failed to implicate the “rogue” middleman/ wholesale distributor in drug offenses which created doubt and gave benefit of the doubt to the accused, of substandard and falsified drugs.

The “closed” and secure drug supply chain is the solution to the problem. The study revealed the modus operandi of middleman/wholesale distributors was that they got spurious drugs from unlicensed manufacturers and mixed them with quality drugs provided by a licensed manufacturer/importer. Licensed manufacturers are unlikely to indulge in manufacturing spurious or substandard drugs deliberately, hence, this was conducted by peddlers or middlemen. Such dubious exercises are difficult to detect by regulators due to poor understanding of the law and connivance of drug regulators with the drug law offenders.

Moreover, the situation is grimmer over here as there are several unauthorized middlemen involved in the drug supply chain in our country besides the fact that a major portion of the business of medicines is in the hands of uneducated people and only 5% of pharmacists are available at pharmacies [47]. Similar findings have been found in the literature that medicines pass through multiple transactions, going back and forth, before reaching the supply point.

The risk of counterfeit, substandard, spurious medicines reaching patients increases with the increasing length and complexity of the supply chain [22,23,48]. It is pertinent to mention here that similar issues were faced in the USA, and they promulgated the Drug Quality and Security Act 2013 and the Drug Supply Chain Security Act 2013 which had to be fully implemented in ten years to address the issue of supply chain. This issue was addressed in the USA by the enactment of the Drug Supply Chain Security Act on Nov 27, 2013, and similar legislation is also warranted in our circumstances [49]. Similar views were expressed by Peter J. Pitts, former FDA associate commissioner, in an article wherein it stressed the need to secure the pharmaceutical supply chain and suggested that counterfeiting can be reduced significantly through product serialization to trace the physical movement through the supply chain using tracking information [45]. Bar code anti-counterfeiting technology could be adopted at least initially for high-priced life-saving drugs to ensure product integrity in the entire supply chain, in addition to the solutions already mentioned in this paper.

Results were endorsed by one study which revealed that wholesalers may sell their medicines to other wholesalers to cover temporary shortages or to reduce overstocked items. It is common in the pharmaceutical supply chain for medicines to pass through several transactions before reaching their destination. This variety of transaction activities allows spurious drug manufacturer to introduce their fake medicines into the supply chain as mentioned in the literature. Our study affirms that the invader in a legitimate supply chain, consisting of primary and secondary wholesale distributors, with the help of peddlers/hawkers/ medical representatives, are involved in the sale/purchase of substandard and spurious drugs [22,48,50,51]. A study by Yakubu and co-workers suggested that loopholes facilitating the infiltration of counterfeit drugs are that the number of unqualified workers in the supply chain is greater than the number of qualified workers, the presence of open markets, and poor implementation of laws [52].

The regulators were duty-bound to secure the supply chain to prevent substandard and falsified drugs from entering. This is one of the real challenges that demands a concerted and systematic approach to curb the menace and amendment in requisite laws and rules/regulations. The situation is compounded in our country as the trail of the sale/purchase of medicines cannot be traced due to non-documentation of transactions and cash basis of payment instead of cheque system. The warranty (pedigree for track and trace) is often disowned by the supplier of substandard and falsified drugs and no documentary evidence is presented before any regulatory body against criminals. In such circumstances, the traceability of questionable drugs is not possible.

Corruption in regulatory authorities is one of the major factors impeding the enforcement of laws effectively in LMIC. It was reported in a study that officials take bribes to allow criminals to continue their unlawful trade [53]. WHO advised member countries to form a task force and fight against the corruption that stops the law from being enforced [54,55].

Defining counterfeit and spurious drugs has been considered a big challenge in combating CSSA drugs due to the lack of standardized definitions for such drugs [11,12]. The definition of counterfeit relates to intellectual property concerning copyright and trademarks [11]. Whereas the term spurious is more prevalent and widely used in our context. The term counterfeiting lies at the intersection of three different streams of law, i.e., Intellectual Property laws, medicines law, and criminal law [56].

The study also highlighted many shortcomings in the drug testing laboratories reports which gave benefit to the accused and resulted in their acquittals in the courts. The study pointed out glaring errors in the reports of government analysts which could not be used as evidence against delinquent pharmaceutical firms. It was pointed out that one of the five laboratories in Punjab tests approximately 40000 drug samples per year and all five laboratories test roughly 200000 samples, mostly of public sector tender business medicines which were made available free of cost for the general public, instead of risk-based random sampling from field. It jeopardizes the rational sampling and quality of testing, contrary to the best international sampling and testing practices, prevailing in the developed world. Moreover, a large number of reports of public sector drug testing laboratories of our country declaring poor quality medicines are inconsistent, vague, and full of flaws in which a large portion of samples tested were from the public sector tender medicine procured through competitive bidding, instead of risk-based random sampling [40]. The Study also identified inter-lab variation of reports of drug testing laboratories and appellate laboratories which gave the benefit of the doubt to the accused firms and frustrated the very purpose of drug laws. Many reports issued by the laboratory are time-barred and without any further extension, losing their legal value in a court of law. The study also raised serious questions on the transparency of these reports and blamed the government analyst for showing efficiency by issuing substandard reports by misusing authority. The reports of the appellate laboratory also raised many question marks. To resolve this problem there is a need to change the policy and appoint highly qualified trained, and experienced pharmacists/ Chemists, etc., in the laboratory so that the reports produced are credible and can be used in interdicting the manufacturers of substandard and spurious drugs effectively.

The research showed that scrutiny of drug cases at Quality Control Boards was often compromised. The cases are often decided without the application of technical and judicial minds. The Board failed to implicate the rogue wholesaler/distributor as accused of drug offenses and miserably failed to ascertain the names of those prima facie responsible for an offense due to lack of technical/legal acumen and maybe for extraneous considerations. It was suggested by one of the participants of the research that PQCB must be made independent and no employee of the health department should be its member under the chairmanship of a retired judge of the High Court. This suggestion is worth consideration to ensure merit-based unbiased decisions free from any technical and legal infirmities.

The study found lacunas in the drug cases resulted in acquittals of accused/culprits, as cases were not properly investigated by the drug inspectors and police etc. Collection of evidence and fulfilling all the legal formalities is a prerequisite for conviction, which was often lacking in drug cases. Drug inspectors often failed to ensure, lawful gazette notification and the mentioning of date, time, and storage conditions like the temperature at the recovery memo at the time of the raid. One key informant rightly maintained that 90% of cases could be acquitted by the drug court due to mere lacunas and material deficiencies in these cases if the full-fledged trial was conducted [57]. One key informant justifiably suggested that the chairman of the drug court should be a retired judge of the high court and members must be independent without any link with the executive to make the Drug tribunal, vibrant, independent, and effective.

In a media report, it was also highlighted as an alarming situation that drug courts handed down minor punishments to the accused despite the recovery of substandard and spurious drugs. The High Court took it seriously in revision jurisdiction and declared that imposing petty fines is just like punishment awarded for a traffic violation or some price list violation [58] as a study conducted in India identified various impediments to the implementation of the Act. Ambiguity regarding the regulations of dietary supplements, Indian Systems of Medicine & herbal products, and ayurvedic cosmetics as the probable reasons that deter the effective implementation of the Act [59]. The finding of the study of the availability of more substandard drugs compared to spurious/counterfeit drugs is corroborated by Helen M Sammons et al [60]. The study found that posting honest officers in field and drug testing laboratories having higher qualifications and requisite experience, on merit, was essential to effectively implement drug laws in the country and to produce credible reports to be used as conclusive evidence of facts stated against culprits.

## Limitations

As the study was mainly conducted in Lahore, the results cannot be generalized to represent the rest of Pakistan. Moreover, the response failure from the participants is an inherent limitation. All the participants had varying degrees of experience and knowledge, and there was also a difference in their opinions at some points covered in the study. and the variation of views of participants represented the study’s strength. Drug Testing/analysis data of public sector Drug Testing laboratories in Punjab could not be considered representative due to inherent sampling flaws as a major chunk belonged to Tender business medicines procured in public hospitals, instead of risk based random probability sampling and real prevalence of Substandard and falsified drugs could be higher. The views expressed by drug law experts may be biased in some places due to the conflict of interest and their personal experience as they represent pharmaceutical firms to get relief for their clients.

## Conclusion

Substandard and falsified drugs are an understudied problem in Pakistan and require concerted efforts to eradicate this menace. Drug control infrastructure, political will, and vision of the government, proper drug monitoring, enforcement of the law, securing the drug supply chain, ensuring the presence of pharmacists at pharmacies, enforcement of valid warranty (track and trace) system, documented medicine sale/purchase and payments through cheque and training of regulators including all stakeholders can help tackle this problem.

## Prospects and recommendations

In the future, more studies can be performed on this issue by considering samples from all over the country and including more stakeholders including physicians, pharmacists, and distributors for a comprehensive understanding of the issue. The study recommends the implementation of the drug laws of Pakistan in letter and spirit to combat the issue. This study can be used as a reference to ensure the integrity of the Drug Supply Chain which is the crux of the study. We also recommend that further in-depth study of the drug supply chain issues in Pakistan be carried out for policymakers to curb the menace of SF drugs.
